# Global truffle market: Theoretical aspects and empirical evidence

**DOI:** 10.12688/openreseurope.19640.1

**Published:** 2025-03-03

**Authors:** PABLO PODADERA-RIVERA, FRANCISCO J. CALDERÓN-VÁZQUEZ

**Affiliations:** 1 Facultad de Ciencias Económicas y Empresariales, España Universidad de Málaga (UMA), Málaga, Andalusia, 29013, Spain

**Keywords:** Global markets, truffles production, truffles trade, rural development

## Abstract

**Background:**

The truffle has gone from being a culinary singularity of certain Mediterranean regions (France, Italy and Spain) to becoming in recent decades a luxury commodity in the agricultural markets with high prices and high profitability for producers and suppliers. This truffle’s centrality generates a constant rising demand that provokes new suppliers to try to enter this market within the framework of globalization. Until now, studies about truffles have focused mainly on physical, biological and agribusiness aspects.

**Methods:**

This study proposes a descriptive analysis of the global truffle market. The analysis starts with truffle production, focuses on the factors that determine it, and has a direct impact on the supply. To analyse the interaction between supply and demand for truffles, we have generated two double-entry matrices where the ten largest importers and the ten largest exporters are analysed in relation to prices and quantities imported and exported. To this end, we have studied the factors determining production and prices of truffles, as well as the interrelations in the global market and the added social and ecological value of their cultivation.

**Results:**

The fundamental result of the analysis is a three-dimensional and holistic image of the whole market that reflects both the interacting agents in terms of production and trade, import and export, and product flows in the world.

**Conclusions:**

Truffles are not only important for their commercial and market value, but also for their environmental and social values. Socio-demographic and environmental values of truffles their cultivation that make truffle production a driver of agricultural and rural development, as the experience of certain rural and continental areas in the European Union evidences. These values are particularly important because they configure the truffle as a community commodity whose production can benefit both the rural population and territory in question.

## Plain Language Summary

Globalisation brings a new context that implies a new way of thinking and acting, which makes it necessary to carry out specific analyses of certain unique products from a global perspective. As regards the truffle, this so-called "cookings diamond", its trade has seen a global boom in recent decades. This justifies the need to carry out analyses that highlight the importance of this product in global trade, not only from a commercial perspective, but also from an environmental and social perspective, as well as its contribution to agricultural and rural development, to the demographic support of rural areas, as well as to the valorisation of the rural environment.

## Introduction

In an increasingly interconnected world, the global market has become a complex network of commercial exchanges that transcends borders, cultures, and economies. This vast system not only facilitates the flow of goods and services but also drives innovation, competition, and economic development across diverse regions. From technological products to exotic foods, international trade allows consumers access to a variety of options that enrich their lives and experiences.

Within this context, the global truffle market stands out as a fascinating and exclusive niche. Truffles, known as “cooking’s diamonds,” are a type of underground fungus that grows in symbiosis with certain trees and has been valued for centuries for its unique flavor and aroma. As demand for gourmet products has grown, interest in truffles has increased, making them a luxury product in global gastronomy.

This market, although relatively small compared to other agricultural sectors, shows unique characteristics that deserve a detailed analysis. From production and harvesting in specific regions of Europe to the growing popularity in emerging markets, where global supply chains, technological globalization, trade liberalization, international marketing, and strategic alliances play a major role, the truffle trade reflecting not only culinary trends, but also the economic and environmental dynamics that affect global agriculture.

In this context, it is essential to explore both the theoretical aspects underlying the truffle economy, such as supply and demand, competition, and sustainability, and the empirical evidence supporting these theories. Through a detailed analysis of production data, prices, and consumption trends, a clear view of how this market operates and adapts to changes in the global environment can be obtained.

Therefore, the aim of this study is to analyze the theoretical aspects that govern the global market in general and the truffle market in particular, as well as the empirical evidence showing the growth and challenges of the truffle market, providing a comprehensive perspective on how this gourmet product fits into the international commercial landscape.

The remainder of this paper is organized as follows. After this introduction, a section focuses on some of the theoretical aspects as well as relevant literary contributions in this regard, which underlie the globalization of markets today.

The following section develops a descriptive analysis of the global truffle market by studying the conditioning factors of elements such as production and its social and ecological value, price, and global trade. Finally, a final section is included, and conclusions on the subject are discussed.

## Theoretical background. Market globalization. Key elements

The literature on market globalization is quite extensive and diverse, depending on the subject and/or approach that one wants to highlight. Thus, we find contributions that emphasize the impact of globalization within the framework of the fundamental principles of economics, with J.L. Sampedro (2010) as one of its outstanding representatives, or those that focus on marketing, highlighting, to this end, the need for market practices to be extended globally, thus allowing large companies to internationalize through the use of operational and marketing practices in the countries where they sell their products ( Levitt, 1983) ^1^.

But also in the current context of openness and globalisation of the economy at a world level, taking advantage, above all, of the emergence and consolidation of technologies, SMEs play a fundamental role as decisive elements of competitiveness, both at a national and international level. That is why they also need to take and adapt to a series of elements that the globalisation era introduces to the competitiveness of SMEs and their internationalisation ( Calof & Beamish, 1995; Welch & Loustarinen, 1988; Welch & Welch, 1996), in a more gradual or more automatic way (the so-called “born-globals”, among many other similar terms). Hence the emergence, in recent decades, of scientific research on business internationalisation as a key factor in their marketing strategies ( Johanson & Vahlne, 1977; Johanson & Vahlne, 1990; Rialp & Rialp, 2001; Werner, 2002).

In this sense, scientific literature has proliferated in these decades on studies related to the organizational structure and competitive strategies of SMEs to adapt to the new environment of globalized markets ( Camisón Zornoza, 2003; Ebben & Johnson, 2005; Miles *et al.*, 2000, among others), in which the technological and innovative boost becomes essential ( Coduras Martínez, 2010)

In this context, it is necessary to highlight a series of essential elements in the globalization of markets.

We can consider the global market as a category of the globalization process that, like the existence of a global product that is universally consumed and that, therefore, uses productive capacities without restrictions, justifies the scale economies.

But this situation requires global companies to have efficient supply chains, in the sense of meeting consumer expectations in terms of prices and product delivery times in terms of profitability, in the sense of cost reduction. This increasingly requires that companies, in a world of global markets, become part of global supply chains ( Horner, 2016; Montanino *et al.*, 2021; The Economist, 2021; World Trade Organisation, 2019, among others)

Factors such as trade liberalization processes that reduce tariffs and other trade barriers through both bilateral and multilateral agreements, free access to new technologies and the Internet of Things (IoT) that provides global connectivity (technological globalization) or large international infrastructure investment projects, such as the Global Gateway Strategy in the EU ( European Commission, 2021), as well as the optimization of international transport (global logistics), become key elements that favor the globalization of markets.

All of the above also requires that companies get involved in activities at a global level, which requires the search and definition of strategic alliances ( Arranz *et al.*, 2016; Cao & Zhang, 2011; Mora-Castellanos *et al.*, 2019, among others) in the globalized environment. For its part, in the previously defined context, International Marketing Strategies in global markets become essential tools when approaching said markets, where strategic marketing, analysis of international markets, technological innovation, international experience, or the social, political, cultural or legal environment itself, they are configured as fundamental factors when approaching them ( Bouncken *et al.*, 2015; Chen *et al.*, 2016; Czinkota & Ronkanien, 2013; Ilayda, 2021; Keegan & Schlegelmilch, 2001, among others). Finally, since this work focuses on the global market for truffles, considered a luxury food product, it seems appropriate to highlight the peculiar case of the internationalization of SMEs specialized in luxury food products in global markets, since these are products with specific characteristics of production, territoriality, quality, craftsmanship... all of which determine their prices, their social and ecological value or their global inherence.

This has sparked interest in research on the international marketing strategies of luxury small and medium-sized enterprises (SMEs) in global niches in which market selection, entry modes or the international marketing mix in foreign markets become prominent variables ( Milanesi *et al.*, 2020; Milanesi *et al.*, 2024).

## Method

## A descriptive analysis of the global truffle market

### Factors determining current truffle production

We defined truffles ^2^ as a type of hypogeous or subterranean ^3^ fungi that normally live in association with the roots of certain trees, especially of the Quercus genus (such as oak or holm oak), with which they establish a symbiosis (mycorrhiza) from which both the fungus and woody plant benefit. Truffles were found below the surface, at a depth of approximately 20 cm. There are more than 70 different species of truffles, with around 40 different types of truffles in Europe, of which the most valued in cooking for their aroma and flavour are Tuber melanosporum (black truffle), Tuber aestivum (summer truffle), Tuber magnatum (white truffle) and Tuber uncinatum (autumn truffle).

Since the 19th century as "diamond cooking" ( Brillat-Savarin, 1838), truffles have historically been used in Europe as a "premium gourmet" ingredient with numerous culinary and gastronomic applications, qualities that in the global world have made it a commodity ^4^ of high global demand and high prices, very rewarding for suppliers (producers, intermediaries, and sellers).

This gastronomic and culinary truffle *centrality* derives from its status as a flavor and aroma enhancer, since with just a few grams it greatly enhances the flavour/aroma of the most varied culinary recipes, from a simple scrambled egg to the most sophisticated haute cuisine dish ( Etxarri, 2023)

Because truffles are grown and harvested in peculiar natural environments normally endowed with specific microclimates for only a few months a year, their wild production could be affected by numerous external agents, mainly climatic (droughts, frost, etc.), and not only (attacks by rodents and animals such as goats, wild boars, rabbits, etc.). Therefore, neither the quantities of truffle to be produced nor their final quality are guaranteed.

Given this random component in its wild production, the imbalance between Supply and Demand for truffles can be on the order of the day, as there are significant levels of uncertainty in its production process. Hence, the tendency for the kilo of truffle to reach (as in fact happens periodically) exorbitant prices, since the existing imbalance between a limited supply (due to its scarcity, especially in the most appreciated varieties such as the white truffle ^5^) and a growing demand for truffles (in all its varieties), has always been increasing since the end of the seventies.

Similarly, a decline in wild truffle production was observed. The decrease or stagnation seems to be due to various causes such as overexploitation, pollution, loss, and deterioration of natural habitats. On the other hand, the climatic conditions of recent years and frequent droughts in the Mediterranean arc generate a natural context that is not very favorable to wild production. Hence, there is a tendency to replace wild production with artificial crops (plantations) to satisfy growing demand.

In short, faced with increasing demand (and the resulting high market prices), given the impossibility of obtaining more wild production, suppliers and producers have chosen to introduce its cultivation in Europe, with Spain and France being the largest producers of Tuber Melanosporum for plantations. Although such plantations are not exempt from problems either because truffle-producing areas require certain soil conditions, quality humidity, and heterogeneity of the plants sine qua non, the area suitable for its production cannot be just any ( Hall *et al.*, 2007; Morcillo *et al.*, 2024; Olivera *et al.*, 2011).

This makes their production even more difficult given the current climatic trends, either due to increasingly frequent periods of drought and intense heat or irregular rainfall, which has led to an increasing recourse to irrigation and the implementation of irrigation systems and devices in production areas. Finally, the production of cultivated truffles has a significant period of deficiency in terms of investment (and recovery of the same), since, on average, the production of truffles is not usually significant until the tenth or twelfth year after sowing ( Olivera *et al.*, 2011), reaching full production at twenty or twenty-five years, a boom stage that lasts about ten years, beginning the decline at 30–35 years, and reaching its practical end at 40–45 years ( Fertiberia, 2016).

Such production restrictions have a direct impact on production variability, meaning that fluctuations in the final price to the buyer can be a constant and significant element of remuneration for the seller.

### Factors determining the price

Today, truffles have become a sought-after commodity. This growing demand has meant that supply has not been able to respond with the same dynamism, which has caused an increase in prices.

As we have seen previously, both the interaction between the supply and demand of truffles and the definition of the price are conditioned by a series of limiting physiological factors (climate, soil, altitude, rainfall, etc.) and management factors (suitability of the site, planning of the intervention, know-how, irrigation, pruning, host trees, pollutants, etc.), to which we should add aspects, such as the type of truffles in question and the locality of the main truffle markets.

Regarding the former, both supply/demand tensions and the resulting price will depend greatly on the variety of truffles in question. Thus, if the chosen variety were the black truffle (melanosporum), due to its high culinary value, its organoleptic properties (intense aroma, etc.), the scarcity of the product and its exclusivity, prices on the market can rise up to €1,000 per kilo ( Acebal, 2022), although according to other sources, in Spain it is usually around an average of €400/kg, with truffle growers charging between €200 and €850 per kilo of unselected dirty black truffle ( Morcillo *et al.*, 2024).

On the other hand, the Summer Truffle (season from May to September) or the Autumn Truffle, also known as the Burgundy Truffle (harvested during the months from September to November), both have a much milder aroma and flavor than the melanosporum, which makes it less valuable to buyers, resulting in a much more affordable price than the Black Truffle.

Undoubtedly, the variety that commands the highest prices is the white Alba truffle (Tuber magnatum). Unlike black truffle, this variety has never been cultivated. Because it is a wild variety, it can only be harvested. It appears only in several regions of Italy, small areas of Croatia, and Bulgaria. For all these reasons, its price can reach 6,000 per kilo ( Acebal, 2022).

With regard to what we previously called localism of the markets, the definition of the price will not only depend on the variety of truffles selected, but also on the place (zone, town, region, country) where it was produced will be very significant. Thus, the average price of truffles in wholesale markets in recent years has been 384 euros/kg in Spain and 535 euros/kg in France (in general, French prices are approximately 40% more expensive than Spanish prices) ( GGM, 2024; Jara Taito, 2016). It should be noted that the interaction between buyers and sellers of truffles is atypical in terms of places, times, and transactions. Points of purchase and sale are characterized by their opacity, which is sometimes similar to clandestine markets full of shadows and uncertainty. Trade is conducted between producers and intermediaries who buy exporters.

Finally, stocking is difficult because fresh truffles are perishable products that must be sold weekly to prevent depreciation due to dehydration, and they must be stored in a cool, dry, and dark place. These elements are considered in truffle production management.

In any case, recent trends in relation to truffle prices indicate that there is still a lot of market to satisfy, since although the offer has increased in recent years, prices have continued to rise, proving the current gastronomic centrality of the truffle. Currently, the estimated value of the global truffle market exceeds $ 400 million. This value increases progressively each year, with a compound annual rate of 7.92%, reaching almost 600 million dollars in 2029 ( Businesscoot, 2024).

### The added values of truffle production: social value and ecological value

Sometimes, we could not see the forest for trees. In the case of current truffle production, the high prices and existing and potential profitability have placed truffles in a relevant position within the global agricultural panorama. However, in addition to its economic value, as a commodity, other values appear in the European truffle experience that should be highlighted: On the one hand, we could consider the truffle as a social or community commodity, in English a “community commodity” since it is a primary product that requires and involves a large number of individuals, families and small and medium-sized enterprises (SMEs) in its production process.

To cite a few examples, in the Spanish experience, Sarrión ^6^ (Teruel), Villaciervos and Abejar (Soria), Vistabella del Maestrazgo (Castellón), Graus and the northern area of Huesca, Centelles and Vic (Barcelona) stand out, which together constitute personifications of that community based on truffle production, which in turn sustains the prosperity of these territories. In Italy, municipalities such as Pietra Lunga and Norcia (Umbria), Alba and Langhe (Piedmont), San Miniato (Tuscany), Acqualagna, and Sassoferrato (Marche) are other examples of this triumphant triangle of truffle culture, truffle production, and comparative prosperity.

Since truffle plantations are operated with trees that are mycorrhized with truffles, truffle production generates forest patches that encourage the afforestation of agricultural land with native trees from the Mediterranean basin, contributing to biodiversity through the creation of new forested areas ( Olivera *et al.*, 2011) and greater sustainability of land use ( Eichhorn *et al.*, 2006). On the other hand, they enhance the value of soils that would otherwise not be profitable for their owners, as occurs in many continental rural areas across the European map. In any case, it is an agroforestry and ecological crop whose contribution to the development of rural areas with scarce resources has been clearly proven ( de Román & Boa, 2024; Reyna Domenech & García Barreda, 2012; Sáez & de Miguel, 2008). Finally, the “savoir faire” involved in the cultivation and production of truffles contributes to the revaluation of rural areas and the agricultural environment. Not everyone knows how to produce truffles, and although the progressive expansion of truffle plantations to other areas of the world contributes to the standardization of this knowledge, the original know-how transferred intergenerationally continues to be another asset of rural heritage.

## Results and discussion

Tuber melanosporum, known as the black truffle or the Perigord truffle (southwest of France), is not found all over the world, only in some regions where the soil and the environment are favorable. Therefore, it is only collected in the wild or naturally in European countries with Mediterranean climates and specific weather conditions ( Dossier Tecnic, 2008). All world production of wild (non-cultivated) black truffles comes entirely from Europe, with Spain, France, and Italy being the main producers of wild black truffles obtained from their native forests.

The most important regions for collecting (cultivating) black truffles were Spain. The areas with the highest production ^7^ are Teruel, Lleida, Huesca, Cuenca, Valencia, Soria, and Tarragona, among other provinces in the northeast of the country ( Etxarri, 2023).

In France, black truffles can be found in areas of Provence and, in general, the southwest is a very important production area; Drôme, Vaucluse, and Alpes de Haute Provence are the three main regions where tuber Melanosporum truffles are harvested in the wild ( GGM, 2024).

Italy is home to important truffle-growing areas in the regions of Piedmont (Alba, Langhe, and Alta Val Bormida), Umbria (Norcia, Spoleto, Città di Castello, Pietralunga, and Gubbio), Tuscany (Crete Senesi, San Miniato della Val Tiberina, Mugello, Casentino, Chianti, etc.), and the Marche region (Acqualagna and Pergola, Sassoferrato, Fabriano, etc.). In the case of Italy, although more important, production is the existing truffle culture, both for aspects such as harvesting (among wooded hills, by hand, and with trained dogs) and for the wine and food link and culinary excellence that links the truffle and the continental rural world with the outside, enabling a much weaker tourist and leisure link in other European countries. Italy is also a world leader in trade and the generation of value from truffles ( Businesscoot, 2024).

The annual production of Tuber melanosporum in Europe is estimated to be approximately 50 tons. Spanish production is stable at an average of 22 tons per year, with values between 10 and 30 tons depending on the climatic year, representing between 30 and 40% of the world production. While truffle production at the beginning of the century in France reached 1,000 tons per year, it barely reached 20 tons per year in the 1990s. Spain ^8^ produces more than 40% of the world's plantation truffles, with the autonomous community of Aragon led by the province of Teruel, being the largest producer at the national level, producing 30% of the world's plantation ^9^ black truffle production.

The high prices and, consequently, the high profits generated by the truffle have sparked enormous international interest in the truffle, its cultivation, and production. Hence, new producing countries arrived at the international truffle market in the 1990s, such as the United States, New Zealand ^10^, Australia, Argentina, Chile, ^11^ and South Africa, among others, and countries with a temperate Mediterranean climate.

Globalization, as in many other areas of activity, is changing the traditional physiognomy of truffle production, with the appearance of new producers, new commercial flows, and new exporters and importers, as shown in Table 1 and Table 2, prepared from records in the Tridge database (2023) based on the HS code 070959 (which is the representative HS code for the truffle) (2023).

**Table 1.** Top ten Exporters/Producers according TRIDGE DATABASE.

COUNTRIES 2021	GLOBAL EXPORTS %	VALUE $ M	GROWTH IN EXPORT VALUE 2020-21	GROWTH IN EXPORT VALUE 2018-2021
**CHINA**	**28.04%**	**270.14**	**+15.74%**	**+38.64%**
**ITALY**	**9.44%**	**90.97**	**+49.79%**	**+23.83%**
**POLAND**	**6.83%**	**65.83**	**+9.27%**	**+25.29%**
**NETHERLANDS**	**6.26%**	**60.33**	**+7.81%**	**-29.35%**
**ROMANIA**	**5.36%**	**51.59**	**+23.94%**	**+21.31%**
**SOUTH KOREA**	**5.18%**	**49.95**	**0,22**	**-0.68%**
**SPAIN**	**4.53%**	**43.62**	**46.17%**	**54.56%**
**LITHUANIA**	**3.73%**	**35.94**	**-5.70%**	**+5.81%**
**BELARUS**	**3.28%**	**31.58**	**+14.71%**	**+9.40%**
**BULGARIA**	**3.05%**	**29.36**	**40.09%**	**11.23**

Source: Tridge Database (2023)

**Table 2.** Top ten Importers/Producers according TRIDGE DATABASE.

COUNTRY	% GLOBAL IMPORTS	VALUE $M	GROWTH IN IMPORT VALUE 2020-21	GROWTH IN IMPORT VALUE 2018-2021
**VIETNAM**	**10.84%**	**106.10**	**+305.69%**	**+497.49%**
**GERMANY**	**9.97%**	**97.60**	**-3.13%**	**-6.76%**
**FRANCE**	**9.8%**	**95.45**	**-**	**-**
**ITALY**	**8.29%**	**81.19**	**+23.90%**	**+9.39%**
**UNITED KINGDOM**	**7.61%**	**74.52**	**+31.97%**	**+16.56%**
**UNITED STATES**	**6.46%**	**63.27**	**44.26%**	**+21.66%**
**JAPAN**	**6.00%**	**58.73**	**+4.70%**	**-2.44%**
**NETHERLANDS**	**4.38%**	**42.86**	**+32.46%**	**+4.18%**
**THAILAND**	**3.13%**	**30.67**	**+3.64%**	**+0.19%**
**SWITZERLAND**	**2.90%**	**28.37**	**11.20%**	**+19.75%**

Source: Tridge Database (2023)

Under normal market conditions, large producers tend to be large exporters, although not necessarily (given the role of intermediation). Likewise, if the Demand for Truffles is closely linked to catering, transformation, and tourism, large consumers will tend to be large importers, either consuming or processing the raw material for the uses mentioned above.

That said, we can see how China, as in many other areas of economic activities, dominates global truffle export flows, becoming the world's leading exporter, with almost 30% of total exports reaching a value of 270 million dollars (2023). This overwhelming amount of data must be sifted through, since, although in recent years total truffle production in China has been around 200 tons, almost half of world production, the trend is to decrease every year, with the provinces of Yunnan and Sichuan meeting the ideal natural conditions for truffle production, with 80% of Chinese truffle production located in these areas. A large part of production is destined for exports, with its main destinations being Spain, France, Germany, Japan, and Holland.

On the other hand, the Chinese truffle variety (Tuber indicum Cooke et Massee) is considered to be of lower quality than the Italian white truffle (Tuber magnatum Pico) and the French “Black Diamond” black truffle (Tuber melanosporum Vitt), which is why it is a producer-exporter flow of low comparative quality, low prices, and high volume. For this reason, large Asian importers tend to import truffles mainly from China (low quality, but cheap and in large volume), compensating for this with flows from Italy and France (lower import volume, but very high quality).

In addition to the omnipresent Chinese presence, the presence of five countries from Eastern Europe (Lithuania, Belarus, Bulgaria, Romania, and Poland), currently integrated into the EU, except for Belarus, stands out among the top 10 exporters. A surprising presence, since they account for 22% of total exports and a not inconsiderable value of more than 200 million dollars, clearly putting pressure on or almost surpassing traditional Western European producers-exporters (Italy, Spain, France, Holland) who, while maintaining their positions as a whole (representing 20.23% of total exports and 194.92 million in value), see how production-exports (both raw materials and processed products) from Poland or Romania reach large production volumes and value, conditioning their former (once, formerly) monopoly.

In Table 2 (10 main importers), there are clear signs of intra-industry trade in parts and components, as in the cases of Italy, France, and Holland, large producers, and exporters that nevertheless need more raw materials and processed truffles to meet their production and consumption needs. On the other hand, it is evident that the major importers and consumers of truffles are Western countries (USA, UK, Germany, Switzerland, and Japan), which together represent 50% of the total volume imported by the 10 major importers, reaching a value of almost 500 million dollars. These countries buy large quantities of truffles to meet their consumption needs (gastronomy-restaurants, culinary, etc.) and transformation (confectionery industries, food preparations, etc.).

Meanwhile, Asian countries represent 20% of the total imported volume and a value of almost 200 million dollars, which symbolizes significant changes that occur daily in the global world. In this sense, the fact that Vietnam is the world's leading importer leaves no doubt about the present and future importance of the truffle business for countries that until recently were part of the so-called Third World and that are now pushing hard to land among developed countries. These countries, which will gradually become increasingly numerous and have a greater share in trade volumes, see truffles as a product with wide margins, whether in its production and marketing (as a commodity or as a processed product) or its intermediation, which is very attractive and can provide certain benefits in an uncertain world.

This market is also characterized by its dynamism, as evidenced by the increase in the value produced in the period (2018–2021), a net increase for both exporters and importers, if we look at both tables. Spain stands out as the exporting country, where the value of exports grew the most between 2018 and 2021, reaching 54%, followed by China (38%), Poland (25%), and Italy (23%). On the import side, Vietnam stands out (+500%), followed by the United States (+22%), Switzerland (19%), and the United Kingdom (16%). As we can see, these upward drives in the demand of consumer-importing countries stimulate greater production so that the supply can provide more products to an unsatisfied demand.

Finally, if we look at both export and import flows in parallel, we see that this is an atypical global market in terms of regionalization. Unlike other commodity markets, where flows are on a global scale between countries belonging to different continental regions, such as the global markets for coffee, wheat, oil, soybeans, copper, and lithium, in the case of truffles, large import-export flows occur mainly within specific continental units. Thus, within Asia, China is the main supplier to Vietnam, Japan, South Korea, and Thailand, while in Europe, according to the records of the Tridge Database, Poland has become the main supplier of truffles to the United Kingdom and Germany, while Romania is the main supplier to Italy. France was the main import destination for Spain and Poland. This does not mean that there are no intercontinental relations, as in the case of Italy, a major supplier to the United States, but they are still of a reduced volume in relation to intercontinental relations.

In the near future, these transcontinental relations will probably grow greatly as the parties involved get to know each other better, and transaction costs will be reduced, making it equally feasible to buy/sell truffles or processed truffles in one place as in another. Currently, intra-regional relations constitute the core of the global truffle market. For all these reasons, we can see this peculiar market as the result of the sum of all the unique local, national, and regional truffle markets, which together make up the great global market, fragmented from its base.

## Conclusions

In this study, we have contrasted the existence of a global market for truffles, a complex network of commercial exchanges that transcend borders, cultures, and economies where global supply chains, technological globalization, trade liberalization, international marketing, or strategic alliances play a leading role. This market, although relatively small compared to other agricultural sectors, has unique characteristics that fully justify this study.

As the common denominator of numerous culinary and gastronomic applications, truffles have become a commodity in the global world with high demand, high prices, and high profitability for the agents involved in the global truffle market. Its added value derives from its gastronomic and culinary centrality and powerful links with catering, the agro-processing industry, and tourism.

This “centrality” of the truffle has meant, in terms of the market, an exponential increase in demand which, logically, has brought about an increase in production. The problem lies in the fact that traditional wild truffle production is unable to meet (owing to its production contradictions: droughts, climate change, rainfall, etc.). Hence, the increase in “cultivated” truffle production, opened up the market to new global producers, and significantly increased production in the original areas (France, Italy, and Spain).

A significant factor in determining the price of truffles is the local nature of the markets, that is, the place of production and the type of truffle in question. This could explain why, despite the expansion of production, growing demand has not yet been met; thus, rising prices have been and are a constant in the functioning of truffle markets. This upward pressure on prices attracts, like magnets, new producers, and suppliers who are striving to place new productions on the global truffle market, surpassing the barrier of 400 million Euros in value and projecting a 50% increase in value by 2030.

Globalization, like many other markets, is changing the traditional physiognomy of truffle production and marketing, with the emergence of new producers, new trade flows, and new exporters and importers, with the emergence of powerful Asian competitors (especially China), Eastern Europe, and America.

Although an increase in emerging transcontinental interactions in the truffle trade is expected to be linked to the reduction of transaction costs in the future, the global truffle market currently appears to be made up primarily of the sum of local markets (not very transparent and fragmented), regional markets, and national markets.

Although the mercantilist vision of the truffle places it as a mere commodity with high prices and high profitability, the European experience also infers the great social and environmental value of truffle production. In this way, the truffle can be considered a Community Commodity given its powerful contribution to agricultural and rural development, to the demographic support of rural areas, as well as to the valorisation of the rural environment.

Likewise, its contribution to environmental improvement, owing to the de facto reforestation that truffle production represents in the territory (Quercus), has a significant impact on the recovery of soils and plant cover.

## Ethics and consent

Ethical approval and consent were not required.

## Data Availability

**The data used has been extracted directly from a primary source: Tridge Database (2023): https://www.tridge.com/tridge-eye/market-brief**
