# Supplementary material for: Global truffle market: Theoretical aspects and empirical evidence
Source: Open Res Eur. 2025 Apr 17;5:57. Originally published 2025 Mar 3. [Version 2] doi: 10.12688/openreseurope.19640.2 (PMC12464532; doi:10.12688/openreseurope.19640.2)
Supplement: Peer review history

# Reviewer report: Reviewer response for version 2

**DOI:** 10.21956/openreseurope.21807.r53276

**Published:** 2025-09-25

**Authors:** Jesus Perez Moreno

**Affiliations:** 1 Colegio de Postgraduados, Texcoco, State of Mexico, Mexico

The observations that I made to the previous version have been taken into account by the authors. Therefore, I have no further comments to make.

If applicable, is the statistical analysis and its interpretation appropriate?

Not applicable

Is the study design appropriate and is the work technically sound?

Yes

Is the work clearly and accurately presented and does it engage with the current literature?

Partly

Are the conclusions drawn adequately supported by the results?

Yes

Are sufficient details of methods and analysis provided to allow replication by others?

Yes

Are all the source data and materials underlying the results available?

Partly

Reviewer Expertise:

Mycology, Economic impact, Truffles

I confirm that I have read this submission and believe that I have an appropriate level of expertise to confirm that it is of an acceptable scientific standard.

# Reviewer report: Reviewer response for version 2

**DOI:** 10.21956/openreseurope.21807.r53819

**Published:** 2025-05-28

**Authors:** Patrick Hatzenbuehler

**Affiliations:** 1 University of Idaho, Twin Falls, ID, USA

The objectives of this study are to provide a description of changes in the global truffle market over the past couple of decades, and specifically in describing shifts in exporting and importing countries and the quantities/values traded. While there is potential for this study to reach indexing quality, I suggest that several significant and smaller issues be addressed prior to indexing.

*Significant issues to address*

- The main issue that I have with this paper is that the data in Tables 1 – 2 are inclusive of other products than truffles and so are not really representative of the trends in the global truffle market. Specifically, HS code 070959 is for “vegetables; truffles and mushrooms (other than genus Agaricus), fresh or chilled”. In my experience with these data, the HS code with one more digit (0709591) is more representative of the truffle market, since they are “truffles, fresh or chilled”. The inclusion of “and mushrooms (other than genus Agaricus)” means that many other types of mushrooms such as shiitake and oyster mushrooms are included. Thus, the data in Tables 1 and 2 are not specific for the truffle market.
- Additionally, my interpretation of the objectives of this article is that the authors aim to provide a description of changes in the trade flows of truffles over the past few decades, but they have not achieved this goal. Reporting changes from 2018 – 2021 as in Tables 1 and 2 is not sufficient for identifying shifts in trade patterns due to “globalization” factors that the authors spend a significant amount of text and literature review on.

*Smaller issues to address*

- I do not think the current title fully captures the objectives of the paper. Perhaps the authors can adjust it to include some aspect of changes in the global truffle market over the past few decades, which is a key theme for the paper.
- There are some typos in the abstract and other parts of the paper. Could use some proofreading.
- It could be clearer in the introduction that the authors are focusing on the “global” truffle market rather than that of specific regions (e.g., Europe).
- For the discussion of “orchard-based” production relative to wild, what is “artificial” (p. 6) about planting orchards?
- Much of the discussion of market size in the text is in Euros, so it is a bit difficult to compare the values in the Tables in US dollars with the values in the text.
- The authors mention “profit” but there is no discussion of cost of production/acquisition, so the profit claim is lacking evidence.

If applicable, is the statistical analysis and its interpretation appropriate?

Partly

Is the study design appropriate and is the work technically sound?

Partly

Is the work clearly and accurately presented and does it engage with the current literature?

Partly

Are the conclusions drawn adequately supported by the results?

Partly

Are sufficient details of methods and analysis provided to allow replication by others?

Yes

Are all the source data and materials underlying the results available?

Yes

Reviewer Expertise:

Agricultural Economics

I confirm that I have read this submission and believe that I have an appropriate level of expertise to confirm that it is of an acceptable scientific standard, however I have significant reservations, as outlined above.

# Response

**Published:** 2025-06-09

**Authors:** PABLO PODADERA-RIVERA

**Affiliations:** APPLIED ECONOMY (ECONOMIC POLICY), University of Malaga, Málaga, Andalusia, Spain

Dear Reviewer, Thank you for the time you took to review our work and for the suggestions you made for its improvement. Below, we attempt to address each of your suggestions. **Significant issues to address**  

- We understand that code 0709591 is more specific to the truffle market, but we deliberately chose code 070959 because, in addition to the fact that the Tridge database we used did not have data from more recent years at the time, we considered it more realistic from a production perspective and was valid for our work. Furthermore, some experts consulted in our field research (as part of the INTACT project, where this paper is developed) agree that this code can be useful for obtaining a comprehensive view of the market, facilitating data collection and broadening the analysis. The more specific code (0709591) is better if you are looking for an in-depth and segmented analysis, which was not our objective. Therefore, this did not pose any problem for us, since our aim has always been to carry out a descriptive analysis of the global truffle market from a theoretical, generalist perspective, rather than an analytical one (which requires longer annual series). That is why we reflect this in the title.

 

- We have never intended to conduct an analytical study of the evolution of market flows "over the past few decades." This requires long time series and perhaps the application of econometric techniques and/or models, such as data panel and others. There are many such studies already.

Our interest has focused on a more qualitative than quantitative analysis, a descriptive and interpretive analysis that seeks to contrast how globalization is rapidly changing (as it has with so many other products) the global truffle market, starting with production, introducing new producers (China, Latin America, Eastern European countries) and forcing the reinvention of traditional producers (France, Italy, Spain), who must respond to new competition with new production methods, for example, irrigated plantations, as is the case with black truffles in Spain. In other words, for us, globalization represents a new productive context that offers opportunities for new and more efficient producers and threats to traditional ones, and everyone must adapt to this new market by generating new import-export trade links. **Smaller issues to address**  

- Understanding that the reviewer did not easily grasp the objective of the paper and its correlation with the title, we could correct it and change it to this one:

  “Global Truffle Market: Theoretical Aspects and Empirical Evidence in the Current Context of Globalization”  

- We would appreciate it if you could point out the typographical errors you have detected; it would be easier to correct.

 

- We think it is clear from the introduction that the paper focuses on the “global” truffle market, when towards the end it states, “Therefore, the objective of this study is to analyze the theoretical aspects that govern the global market in general and the truffle market in particular.” Reference to the global nature of the paper is also made throughout the text. We believe further redundancy is unnecessary.

 

- "Wild" production basically refers to the collection of wild truffles in forest ecosystems by local agents, sometimes assisted by specialized dogs or through other means. Since these fruits are grown and raised in the ground without human intervention, the total final harvest determines market prices. If the harvest is abundant, prices tend to stabilize or fall, while if it is scarce, prices would rise. This extreme natural conditionality together with the effect of climate changes that have been occurring in the Mediterranean basin with the collapse of wild harvesting in France (and to a lesser extent in Spain and Italy) has caused an exponential increase in truffle farms where truffles are planted and harvested by human hands. Although they grow in a natural environment, it is a anthropized crop prepared expressly for truffle production, often resorting to irrigation, phytosanitary care and new production technologies that make possible a fairly predictable, although not always secure, "harvest."

 

- "Profit" must be contextualized within the context of current truffle production, where ever-increasing demand, compared to a progressively increasing supply, is still insufficient to cover all the emerging demand of recent decades, enabling rising sales prices. In this context, "profit" is subjectively understood as an optimistic perception of the possible or potential profitability of their farms (and their labor) rather than an accounting calculation of profit and loss or costs and benefits.

 

- In fact, in our fieldwork, we have observed very few cases of accounting analysis and a myriad of estimates, perceptions, and rough quantifications, always subjective and always optimistic. Probably a cold analysis of the real profitability of many farms (which would take into account elements such as the opportunity cost of the investment and the detailed total costs) would cast many doubts on their profitability, but this calculating analysis is fortunately very far from the dominant ideas and productive horizons of the great majority of truffle farmers who perceive their work as a way of earning a living in the rural environment (or, if you prefer, a way of living) rather than as a business in the strict sense of the word.
- Pablo Podadera-Rivera ( Author)
- Francisco J. Caderón Vázquez (Author)

# Reviewer report: Reviewer response for version 2

**DOI:** 10.21956/openreseurope.21807.r53275

**Published:** 2025-05-07

**Authors:** Sakhr Alhuthali

**Affiliations:** 1 Chemical Engineering, King Abdulaziz University, Jeddah, Makkah Province, Saudi Arabia

Dear Authors, 

 You manuscript has improved but unfortunately could not see the reply to my previous comments although I noticed some corrections. 

 My only comment this time is that the writing can be improved for example be consistent with currency as you sometimes write $ and sometimes as "dollar" which I assume American dollar you mean. same with Euros, be consistent please. 

 The headlines should be a single informative sentence :

 "Theoretical background. Market globalization and key elements" should be combined or rephrased which I highlighted previously. Maybe Theoretical background:  market globalization and key elements or Theoretical background, market globalization and key elements

If applicable, is the statistical analysis and its interpretation appropriate?

Not applicable

Is the study design appropriate and is the work technically sound?

Partly

Is the work clearly and accurately presented and does it engage with the current literature?

Partly

Are the conclusions drawn adequately supported by the results?

Partly

Are sufficient details of methods and analysis provided to allow replication by others?

Yes

Are all the source data and materials underlying the results available?

Yes

Reviewer Expertise:

Desert truffle cultivation.

I confirm that I have read this submission and believe that I have an appropriate level of expertise to confirm that it is of an acceptable scientific standard, however I have significant reservations, as outlined above.

# Response

**Published:** 2025-05-08

**Authors:** PABLO PODADERA-RIVERA

**Affiliations:** APPLIED ECONOMY (ECONOMIC POLICY), University of Malaga, Málaga, Andalusia, Spain

Dear Reviewer,

 The previous revisions were incorporated directly into the revised text, and we informed the editor of this.

 We also appreciate the comment regarding the consistency of the currency name (which will be reviewed) and the reunification in a single sentence: Theoretical background: market globalization and key elements.

 We appreciate again your review of our work.

 Best regards,

# Reviewer report: Reviewer response for version 1

**DOI:** 10.21956/openreseurope.21244.r51734

**Published:** 2025-03-24

**Authors:** Jesus Perez Moreno

**Affiliations:** 1 Colegio de Postgraduados, Texcoco, State of Mexico, Mexico

This is an interesting and original paper, with high relevance related to the market of truffles available in official sources, which I recommend for indexing, after the following modifications, precisions and corrections, are made:

 i) In the third paragraph of the Introduction section, the authors mentioned “… This market, although relatively small compared to other agricultural sectors, shows unique characteristics that deserve a detail analysis…”. Despite the fact that this statement in relatively true, the cultivation of truffles, e.g., in large regions in Spain or Italy; or their gathering in large areas in China has dramatically improve the economical situation of local populations. This should be highlighted, and included in the Introduction, not in Method section as the authors did it.  

 ii) At the end of the Introduction section the objective of the contribution should be clearly pointed out.

 iii) In the first paragraph of the Method section, it is mentioned that “ … truffles … normally live in association with the roots of certain trees, especially of the Quercus genus… “. This is incorrect, because truffles, even European wild truffles frequently establish mutualistic associations with a number of trees including *Corylus, Prunus, Pinus* and *Tilia*. The authors should correct their statement.

 iv) In the Methods section, it is mentioned “… Unlike black truffle, this variety has never been cultivated..”. This is inaccurate, the cultivation of White truffle has already been achieved (Bach C, et al., (2021) [Ref-1]). The authors should correct their statement.

 v) In the Results and discussion section it is mentioned “… in recent years total truffle production in China has been around 200 tons, almost half of the world production…”, this is an incorrect statement, production of the Chinese truffle ( *Tuber indicum*) has been estimated to be around 1,000 tons per year (e.g., Pérez-Moreno J., (2021) [Ref-2]).

 vi) In the Results and discussion section referring to Chinese truffle it is mentioned “… A large part of production is destinated for exports…”. This is currently inaccurate because their is a growing domestic consumption of a number of truffle species, including new endemic species, in China, including gourmet foods, mentioned or illustrated for example in   DOI: Yu, F., et al., (2020) [Ref-3])

 vii) In the Result and Discussion section the author mentioned “… the so-called Third World…”, referring to a group of countries. The term “Third World countries” is currently considered outdated, pejorative and imprecise. Originally, during the Cold War, it referred to countries that were not aligned with NATO (the First World) or the Soviet bloc (the Second World). Over time, it came to be used more broadly to describe nations with lower economic development. More precise and accurate options are for example, low-income or middle-income countries, which is  used by organizations like the World Bank to classify countries based on their economic status; or emerging markets: Sometimes used to describe countries with rapidly developing economies. Using specific terms related to a country's economic status or level of development is generally more respectful and informative.

 viii) In the Introduction section the acronym SMEs is used without mentioning its meaning the first time that is mentioned, not later as the authors did it; and

 ix) All scientific names should be written in Italics, according to Nomenclatural Scientific rules.

If applicable, is the statistical analysis and its interpretation appropriate?

Not applicable

Is the study design appropriate and is the work technically sound?

Yes

Is the work clearly and accurately presented and does it engage with the current literature?

Partly

Are the conclusions drawn adequately supported by the results?

Yes

Are sufficient details of methods and analysis provided to allow replication by others?

Yes

Are all the source data and materials underlying the results available?

Partly

Reviewer Expertise:

Mycology, Economic impact, Truffles

I confirm that I have read this submission and believe that I have an appropriate level of expertise to confirm that it is of an acceptable scientific standard, however I have significant reservations, as outlined above.

## References

[1] : First production of Italian white truffle (Tuber magnatum Pico) ascocarps in an orchard outside its natural range distribution in France. *Mycorrhiza* .2021;31(3) : 10.1007/s00572-020-01013-2 383-388 10.1007/s00572-020-01013-2 33591428

[2] : Global perspectives on the ecological, cultural and socioeconomic relevance of wild edible fungi. *Studies in Fungi* .2021;6(1) : 10.5943/sif/6/1/31 408-424 10.5943/sif/6/1/31

[3] : Edible Mushrooms and Their Cultural Importance in Yunnan, China. In: Pérez-Moreno, J., Guerin-Laguette, A., Flores Arzú, R., Yu, FQ. (eds) Mushrooms, Humans and Nature in a Changing World. *Springer, Cham.* .2020;

# Response

**Published:** 2025-04-03

**Authors:** PABLO PODADERA-RIVERA

**Affiliations:** APPLIED ECONOMY (ECONOMIC POLICY), University of Malaga, Málaga, Andalusia, Spain

Dear Reviewer,

 We deeply appreciate the time you have dedicated to our work, as well as the corrections and suggestions you have made. We have tried to incorporate them all into the main text of the article.

 Your contributions have undoubtedly enriched our work, for which we are very grateful.

# Reviewer report: Reviewer response for version 1

**DOI:** 10.21956/openreseurope.21244.r51741

**Published:** 2025-03-06

**Authors:** Sakhr Alhuthali

**Affiliations:** 1 Chemical Engineering, King Abdulaziz University, Jeddah, Makkah Province, Saudi Arabia

The article covers a broad spectrum of aspects related to the global truffle market. However, the article needs some improvements in clarity and conciseness, grammar and syntax, flow and cohesion. Some specific points of concern are given below:

1. The abstract needs to clearly highlight the purpose of the study and the answered investigated question.
2. The authors looked at truffle export/import data but it seems they looked at three main countries (Italy, Spain and France) so what are the shortages of this study. For example, desert truffles can not be neglected and clearly some less developed countries are not included in Tridge database.
3. English clarity  for examples “rural and continental areas”, “truffles their”,” dynamism“, “From production and harvesting in specific regions of Europe”, “economics, with J.L.” “studying the conditioning factors”, “Finally, a final section is included, and conclusions on the subject are discussed ( result is usually discussed and conclusion is given)”, “gradual or more automatic way”, “when approaching said market“, “and not only“, “Sometimes, we could not see the forest for trees.“, “countries arrived at the international“, “and a not inconsiderable value“, “the definition of the price will”.
4. Define SMEs in page 4 not later, please.
5. “Theoretical background. Market globalization. Key Elements”. These three sentences should be combined to one as a title.
6. “in this context, it is necessary to highlight a series of essential elements in the globalization of markets“ it is better to include same ideas in the same paragraph. In addition, “Such production restrictions” in page 6 should be combined to previous paragraph
7. In many sentences a “This” was mentioned and it was not easy to know what you referred to exactly, be specific when you use this, these, it, they etc.
8. I think there is no need to add “among others” at the end of the references.“
9. Some references are needed in many sentences such as after “ *Tuber aestivum* (summer truffle), *Tuber magnatum* (white truffle) and *Tuber uncinatum* (autumn truffle).“
10. “can be on the order of the day“, what do you mean?
11. “6,000 per kilo” add the currency and be consistent when writing the word or symbol for euro, please.
12. “194.92“ should be rounded to 195
13. “high prices and high profitability” what is the relationship between these two? Are they always positively correlated?

If applicable, is the statistical analysis and its interpretation appropriate?

Not applicable

Is the study design appropriate and is the work technically sound?

Partly

Is the work clearly and accurately presented and does it engage with the current literature?

Partly

Are the conclusions drawn adequately supported by the results?

Partly

Are sufficient details of methods and analysis provided to allow replication by others?

Yes

Are all the source data and materials underlying the results available?

Yes

Reviewer Expertise:

Desert truffle cultivation.

I confirm that I have read this submission and believe that I have an appropriate level of expertise to confirm that it is of an acceptable scientific standard, however I have significant reservations, as outlined above.
